# Surfactant Protein A and Microbiome Composition in Patients With Atraumatic Intraoral Lesions

**DOI:** 10.3389/froh.2021.663483

**Published:** 2021-04-22

**Authors:** Shawn Adibi, Davor Seferovic, Gena D. Tribble, Joseph L. Alcorn, Walid D. Fakhouri

**Affiliations:** ^1^Department of General Practice and Dental Public Health, University of Texas Health Science Center at Houston School of Dentistry, Houston, TX, United States; ^2^Department of Periodontics and Dental Hygiene, University of Texas School of Dentistry at Houston, Houston, TX, United States; ^3^Department of Pediatrics, McGovern Medical School, University of Texas Health Science Center at Houston, Houston, TX, United States; ^4^Department of Diagnostic and Biomedical Sciences, University of Texas Health Science Center at Houston School of Dentistry, Houston, TX, United States; ^5^Genetics and Epigenetics Program, MD Anderson Cancer Center UTHealth Graduate School of Biomedical Sciences, Houston, TX, United States

**Keywords:** humans, pulmonary surfactant-associated protein a, microbiota, oral ulcer, biomarkers

## Abstract

Oral ulcers are lesions that occur due to disruption of epithelial integrity of the mucosa of the oral cavity. Intraoral ulcers are often associated with pain, redness, symptoms of discomfort, and blood hemorrhage. The etiology for many oral ulcers is local trauma, systemic health conditions, or medication; for other ulcers the cause is less clear. This pilot study aims to evaluate the salivary components and microbiome in patients with atraumatic pre-ulcerous and ulcerous oral lesions compared to control individuals, while considering three common risk factors for atraumatic ulcers, smoking, stress, and gender. This study uses matched age, sex, and ethnicity samples from healthy otherwise and oral lesion patients to investigate the changes in salivary surfactant protein A (SP-A) and examines the prevalence and diversity of the salivary oral microflora. The goal is to determine if there are factors in saliva that have the potential to be used as biomarkers for risk of developing atraumatic oral ulcers. Our data show that the average level of SP-A is significantly reduced in female smokers compared to non-smoker healthy females. The average level of SP-A in female oral lesion patients is reduced compared to controls. The microbiome composition is significantly affected by smoking and the level of SP-A. Comparing the control participants and oral lesion patients, there are 16 species of bacteria that are significantly different, and all of these bacteria are significantly affected by smoking and SP-A. LEfSe analysis identified five bacteria that may represent potential biomarkers. This preliminary study demonstrates the potential of the oral microbiome to act as a biomarker for oral ulcer risk and infers potential mechanistic links between risk factors and alterations in innate immune mechanisms such as SP-A levels.

## Introduction

Mucosal membrane, also known as the mucosa, lines various cavities in the body and is considered the first line of communication with the environment. It also provides the first line of defense against harmful microbes and chemical substances, and intact mucosa is a major component of innate immunity [1]. Intraoral lesions are ulcerous conditions of the mucosa that appear with many medical conditions, including autoimmune disorders, diabetes, and Sjogren syndrome [2, 3]. The multifactorial nature of this condition also implicates host genetics, nutrition, stress levels, changes in hormone levels, and immune responses. Oral ulcerative conditions can occur in a spectrum of severity, and are categorized into four grades (I, II, III, IV) based on the severity of the presentation established by the World Health Organization (WHO) [2, 4]. Grade III and IV are the most severe and are classified by the distinct ulcers that form in the intraoral cavity. These ulcers impact the individual quality of life and well-being, making tasks such as eating and drinking very difficult [4].

Severe intraoral lesions in the form of mucositis may occur when patients are being treated for head and neck cancer with radiation or chemotherapy. It is a common symptom in 80% of patients receiving radiotherapy (RT) to treat head and neck cancer and 40% of patients who receive standard doses of chemotherapy [5, 6]. Mucositis lesions are the most common debilitating complication of drug and radiation treatments for cancer patients. They can lead to several medically concerning problems, including pain, nutritional problems resulting from the inability to eat, and an increased risk of infection due to open sores in the mucosa. Thus, severe intraoral ulcers have a significant effect on the patient's quality of life and can be dose-limiting for cancer treatment [1, 7, 8].

The etiology and pathology of grade III and IV intraoral lesions in immunocompromised individuals undergoing organ transplants or receiving cancer treatment are well-investigated [2, 4, 7]. However, the etiology is not clear and even less is known about the pathogenesis in non-immunocompromised individuals with pre-ulcerous or minor lesions [9, 10]. While most oral ulcer studies focus on patients treated for cancer, a limited number of studies focused on the etiology of minor (Grade I or Grade II) intraoral lesions in the general population.

Treatment of oral ulcers with drugs is not trivial and very few drugs are efficacious [11, 12]. Amifostine, a drug that offers some protection against the damage to the mucosa caused by radiation, is approved by the FDA for patients receiving radiation therapy for cancers of the head and neck [11]. Palifermin (Kepivance) remains the only approved ulcer treatment for non-chemotherapy associated ulcers. Palifermin binds to the human keratinocyte growth factor (KGF) receptor found on buccal mucosa cell surfaces. This binding activates a Ras-MapK (Map kinase) signaling pathway which leads to the transcriptional activation of many proteins necessary for mucosal cell proliferation and survival [13, 14]. These treatment approaches are directed at the down-stream sequelae as no other targets for therapy or prevention have been identified.

Surfactant proteins (SPs) are essential lectin compounds critical for innate immune responses at mucosal surfaces. Originally identified in the lungs, surfactant-association proteins (SP-A, SP-B, SP-C, and SP-D) play a role in reducing the surface tension of the air/liquid interface, preventing the collapse of the alveoli and act as pattern recognition receptors as part of the innate immune defense [15]. Pulmonary surfactant protein A (SP-A) and SP-D are also important for regulation of inflammation [16]. Surfactant proteins in the gastrointestinal (GI) tract create a hydrophobic barrier that protects tissues of the GI tract from acid and harmful microbes [15, 17]. While the oral mucosa has been reported to express SPs that are involved with hydration and protection of epithelium [18, 19], the impact of SPs on the composition of the oral microbiome or in the prevention of intraoral lesions has not been investigated. Our published data has previously confirmed that SP-A is present in saliva [20], and this study aims to determine if salivary SP-A levels are associated with the pathology and severity of oral ulcers.

Ulcers in the stomach (peptic ulcers) and GI tract (ulcerative colitis) have a demonstrable association with changes in the local microbiome and inflammatory immune responses and similar to oral lesions, are influenced by many environmental factors including stress, gender, and smoking status [21, 22]. There are a moderate number of studies on the alteration of the microbiome in intraoral lesions, and these studies indicate a shift in the oral microbiome in the presence of oral lesions. Whether these changes occur as a cause or a consequence of the lesions are not known, and the effect of common risk factors such as gender and smoking status have not been studied with a specific emphasis on intraoral lesions and surfactant levels. In this pilot study, we focused on Grade I or II intraoral soft tissue lesions that present with pain and inflammation, and with either a broken mucosal barrier or tissue redness located in the buccal mucosa, floor of mouth, palatal tissue, attached gingiva, oropharynx, or tongue. These soft tissue lesions excluded viral and traumatic lesions, were Grade I or II only, and were otherwise of unknown etiology. Salivary SP and microbiome analysis was conducted to explore their relationship to the manifestation of atraumatic oral lesions, to potentially identify biomarkers to predict risk, and to discover future pathways for research.

## Materials and Methods

### Study Design, Population, and Power Justification

The targeted population in this study consists of individuals with oral mucositis. As a general oral condition as defined clinically, we categorized the intraoral lesions into four grades (I, II, III, IV) based on the severity of oral mucositis presentation established by the World Health Organization (WHO). Oral lesions from grades I or II were accepted into the study. Grade I includes mucosal soreness and localized erythema. Grade II includes mucosal soreness and tissue ulceration, not interfering with normal diet. Both conditions are irritating to patient to inquire solution from practitioner about it. All intraoral lesions that exhibit break down or local inflammation of mucosa were included, such as ulcerative lesions or red inflammatory lesions in the area of buccal mucoua, attached gingiva, floor of mouth, tongue, oropharynx, and palate. Participants were excluded from the study if they were under the age of 18, had extra-oral herpetic lesions, lesions as a result of trauma (micro, macro), a lesion associated with periodontal conditions, or hyperkeratotic lesions due to smoking or traumatic irritation. There was no follow-up period and participants were released from the study after they completed the clinical assessment and provided saliva samples. Based on our previous study of salivary lipid levels in smokers [20], we were able to detect significant differences in SP-A levels in 27 subjects. For this study, 100 subjects were screened using inclusion criteria that the participants must be a patient, student, or employee at UTSD from January 2018 to December 2019. A total of 36 patients met all inclusion data. Data forms consisting of questions related to participant age, sex, race, ethnicity, smoking status, systemic conditions; number of xerogenic medication(s) taken, World Health Organization (WHO) classifications of mucositis (intraoral lesions), and pain levels were collected. Correlations within demographic and health data were identified using cross-tabulation and Chi-square analysis. Statistical analysis was performed with Stat-plus and GraphPad Prism.

### Ethical Statement

This study has been conducted in full accordance with ethical principles, including the World Medical Association Declaration of Helsinki, and approved by the IRB ethical board of the University of Texas Health Science Center at Houston (Approval number: HSC-DB-15-0742).

### Clinical Evaluation of UTSD Patients

A team of two research staff consisting of a clinician and a clinical research assistant examined all participants. The clinician assessed the patient and entered clinically related information on the data form. The clinical research assistant collected, labeled, and stored samples. The Wong-Baker FACES pain rating scale was used to assess the patients' pain level [23]. The clinician also evaluated each subject based on below clinical criteria and assign a score from no susceptibility [0] to severe susceptibility to bruxism [3]: a. Complaining of tooth sensitivity, b. Multiple damaged dental restorations, c. Presence of moderate to severe tooth wear/erosions, d. Presence of tori, torus, or exostoses, e. Complaining of headache on temporal area, f. Positive high level of stress test, g. Waking up in the morning with masticatory muscle pain, h. Tender masseter muscles on 1 kg force digital palpation, i. Tender lateral pterygoid on ½ kg force digital palpation, j. Tender temporal tendons on ½ kg force digital palpation, as routine and standard screening and evaluation of head, neck, and musckeloskeletal conditions of patient. These measures of pain and oral stress were incorporated as categorical metadata for microbiome and SP-A analysis.

### Saliva Collection and Measurements of SP-A

After participants were recruited and informed written consent was obtained, participants were asked to rinse their mouths with 10 ml of 2% solution of citric acid for 30 s, then spit out the solution. The solution was used to stimulate salivary production [24]. After rinsing and spitting citric acid, the researchers collected two 0.5 ml vials of the participants' saliva. Saliva samples were stored on ice then taken and placed in an ^−^80°C freezer.

We measured the concentrations of SP-A in saliva of healthy and oral lesion patients. ELISA was used to determine levels of human SP-A in saliva samples (BioVendor, LLC, Asheville, NC, Cat. No. RD191139200R) as previously described [20]. The demonstration of SP-A in the saliva of females was performed by western analysis. Antibodies specific for SP-A (#sc-13977; Santa Cruz Biotechnology, Santa Cruz, CA) were used to detect the proteins after PAGE. Protein bands were visualized using the ECL Plus Western Blotting Detection System (#RPN2135, Amersham Biosciences Corp, Piscataway, NJ) and quantified on a Storm 840 Phosphoimager (GE Healthcare, Piscataway, NJ). Since saliva is mainly extracellular fluid samples, there is no reliable internal control like beta-actin to be used for normalization. Therefore, the normalization was strictly based on volume and total amount of proteins. Surfactant protein A data did not have a normal distribution, as assessed by D'Agostino and Pearson test, so further data analysis was performed using non-para-metric tests. Significant differences in SP-A levels were assessed using Mann-Whitney test for two groups, and Kruskal-Wallis for three or more groups. Statistical analysis was performed with Stat-plus and GraphPad Prism.

### Microbiome DNA Extraction From Saliva

From the 36 subjects enrolled in the study, 18 matched subjects were selected for microbiome analysis. Saliva from nine patients above the age of 18 and diagnosed with atraumatic oral lesions, and nine healthy patients with matched ethnicity, sex, and age were used as controls. We matched the affected group with unaffected control based on age, sex, ethnicity (demographic) for the oral microbiome experiment. Total DNA extraction was performed with 500 μl of saliva and the total DNA was purified through adsorption to the silica membrane from UCP Mini Columns of the QIAamp DNA Microbiome Kit (Catalogue 51704. Qiagen, Hilden, Germany) following the manufactures instructions. DNA concentration and purity were measured by Nanodrop 2000® spectrophotometer (Wilmington, USA) and Qubit 1.0 fluorometer.

### 16S rRNA Sequencing of Oral Microbiome

After DNA extraction, DNA samples (100 ng−1 μg) from saliva were submitted for 16S rRNA sequencing performed by LC Sciences (Houston, TX, US, https://www.lcsciences.com/). The 16S rRNA was amplified with primers 338F/806R, and the V3-V4 region sequenced on the MiSeq platform for paired-end reads. Raw data was processed to remove barcode and adaptor sequences, and reads were paired. Low quality or unpaired reads and chimera sequences were removed, resulting in a total of 171, 563 reads in 18 samples.

### 16S rRNA Data Analysis

Community diversity was assessed using the Microbial Genomics Diversity Module of CLC Genomics Workbench v20. Operational Taxonomic Units (OTUs) were clustered against the Human Oral Microbiome Database (HOMD) at 98% identity [25], and unmatched were identified by BLAST against the NCBI 16S rRNA database. OTUs from the abundance table were aligned using MUSCLE with a required minimum abundance of 10. Rarefication analysis was done by sub-sampling the OTU abundances in the different samples at a range of depths from 1 to 100,000; the number of different depths sampled was 20, with 100 replicates at each depth. Alpha diversity measures were calculated for observed OTUs, Chao 1-bias corrected, Shannon entropy, and Simpsons Index. Statistical significance in alpha diversity between groups was calculated with non-parametric tests. PERMANOVA Analysis (Permutational Multivariate Analysis of Variance) was used to detect significant differences in Beta diversity between groups, and comparisons were visualized using Principal Coordinate Analysis (PCoA). Beta-diversity measures were calculated using Bray-Curtis. Differential abundance tests (non-parametric ANOVA) on the OTU frequency table were used to identify significant differences in the relative abundances of individual OTUs between groups. Differential abundance analysis values were calculated with correction for confounding factors and were considered significant with FDR *p*-value < 0.05 (false discovery rate corrected *p*-value), and only if the OTU was present in at least two subjects. Potential biomarkers in the microbiome data were identified using the Galaxy iteration of LEfSe, an algorithm for biomarker discovery and explanation that identifies differentially abundant features that are also consistent across subjects in biologically meaningful categories [26].

## Results

### Population Data

Out of 100 screened patients, a total of 36 subjects were enrolled and sampled: 22 oral lesion patients and 14 controls. Controls were recruited to reflect the diversity of the oral lesion subjects. A summary of demographics is shown in Table 1; the median age of the population sampled was 52 years. The sample population was primarily female (69%) and was comprised of 53% minorities; 19% of minorities were Hispanic/Latino. Twenty-five percent of subjects were smokers, 39% had at least one systemic disease, and 33% were taking xerostomia-inducing medications. Seventy-five percent of study subjects had some degree of bruxism as measured by the Bruxism Severity Index (BSI, ranges from 0 as no clinical evidence of bruxism to 3 as most severe type), and 22% had temporomandibular disorders, TMD (Table 2). In our oral lesion subjects, occurrence of lesions was more common in women (*p* = 0.04), and subjects in their 60 s (*p* = 0.0001) as assessed by Chi-square. Intraoral lesions were significantly associated with pain (*p* = 0.002), and 45% of our oral lesion patients experienced pain level of 2 based on the Wong-Baker FACES pain rating scale (Table 2). Interestingly, the degree of bruxism measured by the BSI correlated with the appearance of oral lesions (*p* = 0.01). Only 3 of our 22 oral lesion subjects scored a zero on the BSI. Smoking is not significantly associated with appearance of oral lesions in this small study, but trends toward significance for our subjects in their 60 s (*p* = 0.07) and in subjects with a score >0 on the BSI (*p* = 0.07).

**Table 1:** Demographics of enrolled subjects.

	**All subjects**	**Percent (%)**	**Oral lesions**	**Percent (%)**
**GENDER**				
Male	11	31	5	23
Female	25	69	17	77
**AGE**				
20–30	3	8	0	0
30–40	6	17	2	9
40–50	5	14	3	14
50–60	7	19	4	18
60–70	9	25	8	36
70 and over	6	17	5	23
**RACE AND ETHNICITY**				
African American	5	14	4	18
Asian	6	17	3	14
Caucasian	17	47	9	41
Mixed race	1	3	0	0
Hispanic Latino	7	19	6	27

*Demographics summary of oral lesion patients and unaffected control individuals*.

**Table 2:** Distribution of health indicators.

	**All subjects**	**Percent (%)**	**Oral lesion**	**Percent (%)**
**SMOKING**				
Smoker	9	25	3	14
Non-smoker	27	75	19	86
**ORAL LESION SCALE**				
Zero	14	39	0	0
One	16	44	16	73
Two	6	17	6	27
**SYSTEMIC DISEASE**				
Zero	22	61	11	50
One	10	28	8	36
Two or more	4	11	3	14
**XEROSTOMIA MEDICATIONS**				
None	24	67	14	64
One	8	22	5	23
Two or More	4	11	3	14
**BRUXISM SUSCEPTIBILITY INDEX**				
Zero	9	25	3	14
One	17	47	12	55
Two or more	10	28	7	32
**PAIN SCALE**				
Zero	24	67	10	45
One	2	6	2	9
Two or more	10	28	10	45
**TEMPOROMANDIBULAR DISORDER**				
Zero	28	78	15	68
One	8	22	7	0.3

*Health status summary of oral lesion patients and unaffected control individuals*.

### SP-A Levels

**Figure 1:**
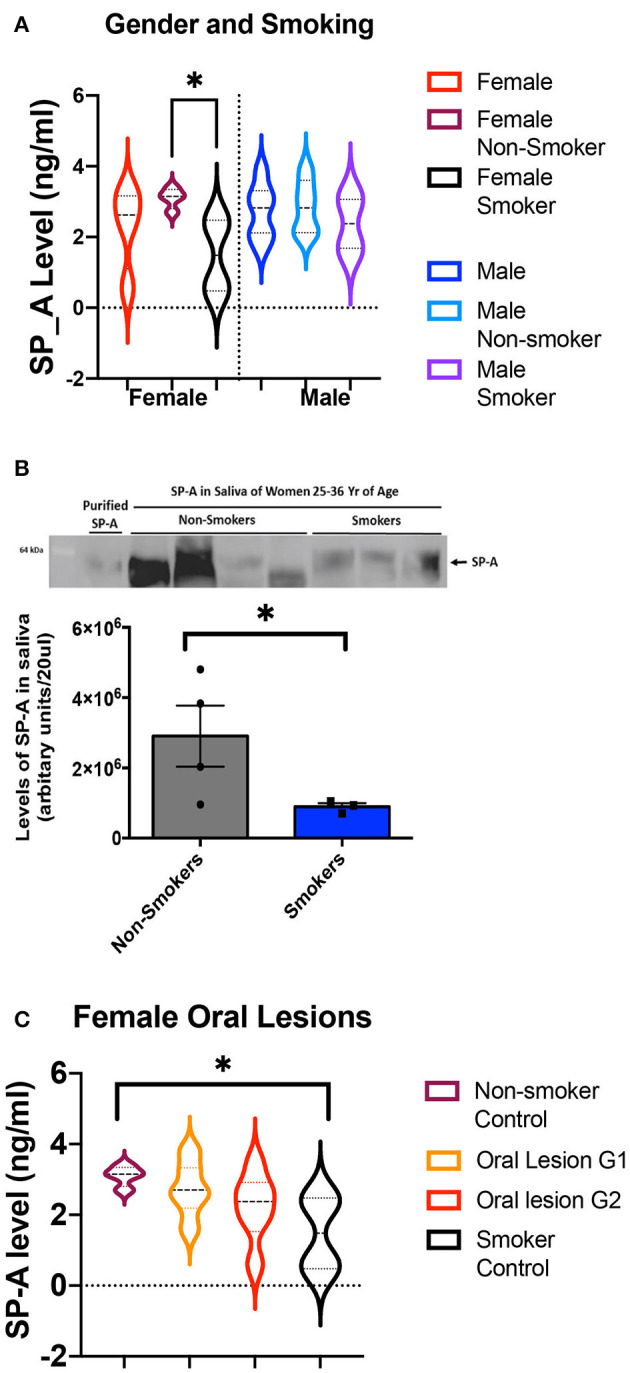
**(A)** ELISA values of salivary SP-A in controls, sub-divided by gender and by smoking habits. The levels of salivary SP-A are shown first for all controls and then split into non-smokers and smokers. Values for female subjects are shown on the left, and male subjects are shown on the right. There are eight female controls, split into four non-smokers and four smokers. There are eight male controls, split into five non-smokers and two smokers. The female non-smokers and female smokers have significantly different salivary SP-A levels (*p* = 0.03) as assessed by Mann-Whitney. No other comparisons were significant. **(B)** SP-A levels in non-smoker and smoker females are significantly different as measured by western blot. The quantification of band intensities is represented below the western blot. An asterisk denotes the statistically significant difference between the samples. **(C)** Measuring the average level of SP-A in unaffected control females and oral lesion patients with grade 1 and 2 severity by ELISA. The oral lesion SP-A values fall between the controls, and suggest a trend in decreased SP-A in females with oral lesions, however the collective Kruskal-Wallis *p*-value was not significant (*p* = 0.09).

Previously we demonstrated that salivary SP-A levels are lower in women than men, and lower in women smokers than non-smokers [20]. This trend continues in this population of 36 subjects, with the level of SP-A in healthy female smokers significantly lower than in healthy female non-smokers. In this study the differences between female and male SP-A levels were not significant, and we did not detect a smoking effect in male SP-A levels (Figure 1A). To verify the differences seen in the ELISA assay, western blot analysis was done and further confirmed that smoking results in a significant decrease in salivary SP-A in females (Figure 1B).

We next compared the levels of SP-A in female oral lesion subjects, compared to the smoker and non-smoker controls. While there is not a significant difference in SP-A levels in intraoral lesion patients, we noted a distinct trend of decreasing SP-A (Figure 1C). With SP-A levels as a possible factor underlying intraoral lesion risk in females, we also assessed if SP-A levels have a significant impact on salivary microbiome composition in healthy control and oral lesion patients.

### Microbiome Diversity

**Figure 2:**
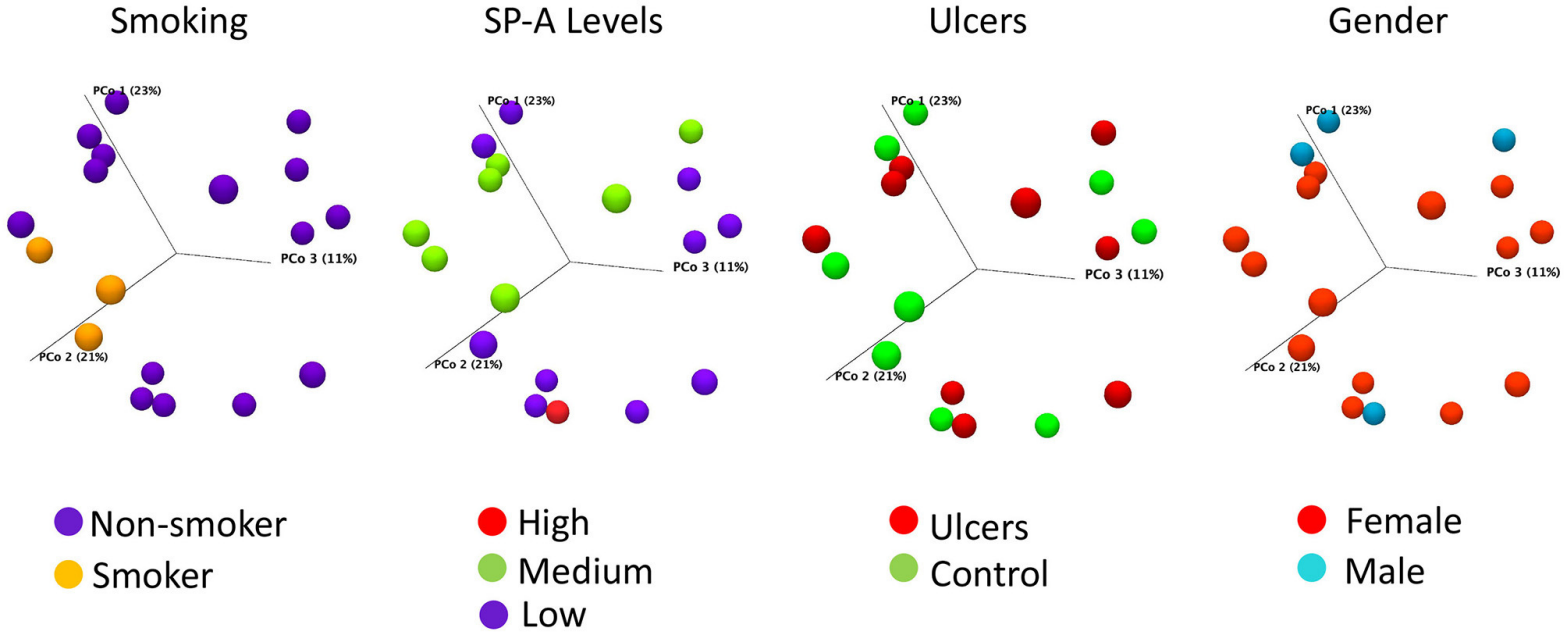
Bray-Curtis PCoA plots of salivary microbiome samples, colored to illustrate the distribution by smoking status, SP-A level, oral ulcer condition, or gender. Axis one accounts for 23% of sample variance, while axis two and three account for 21 and 11% of variance, respectively. Smoking (*p* = 0.03) and SP-A levels (*p* = 0.03) have significant influences on community composition, as assessed by PERMANOVA analysis, while oral lesions and gender do not. SP-A levels were categorized as Low for concentrations between 0 and 2 ng/ml, Average for 2–4 ng/ml, and High for 4–8 ng/ml.

The majority of our oral lesion patients are female and females have a trend of decreasing SP-A; on that basis we selected a sub-set of 18 subjects for microbiome analysis with only one male oral lesion subject and two males' control for reference. Clustering the 16S rRNA sequences from 18 subjects at 98% identity resulted in 383 OTUs, with 244 matched to HOMD and the remaining 139 identified by BLAST. The 383 OTUs when compiled to the species level, represented 249 bacteria present in the saliva of the subset of 18 subjects included in the microbiome analysis. There were no differences in alpha-diversity for oral lesion samples compared to controls (Supplemental Figure 1). Comparing community composition at the level of Beta-diversity, there were no significant differences between subjects with intraoral lesions compared to controls (Bray-Curtis FDR *p*-value 0.96). There were no significant differences by sex (*p* = 0.32) or race/ethnicity (*p* = 0.19). However, we did detect significant effects on the microbiome community using the meta-data points of smoking (*p* = 0.03) and oral levels of SP-A (*p* = 0.03) (Figure 2). Although there are only three smokers in the sequencing arm of the study, this significant result is consistent with larger studies that demonstrate oral dysbiosis induced by smoking [27].

### Microbiome Abundance

Comparing the community composition at the level of bacterial species abundance, 16 species are present in at least two subjects and are significantly different in the oral lesion group compared to healthy patient group (Table 3). However, when you adjust for the confounder of SP-A level on microbiome abundance, seven OTUs are no longer significant (Table 3 Block 1). Repeating the differential abundance analysis adjusting for smoking, *Bifidobacterium dentium* is no longer significant (Table 3 Block 2). Adjusting for both smoking and SP-A levels removes the final eight OTUs from significance (Table 3 Block 3). When the differential abundance species lists are considered collectively, of the 16 bacterial species initially associated with intraoral lesions, all are more strongly influenced by smoking or SP-A levels. Surfactant protein A has a strong influence on individual species levels in this study, with 53 species significantly different, and 35 having an FDR adjusted *p*-value < 0.01 (Table 4). Notably, *Corynebacterium argentoratense* is the most elevated OTU under low SP-A conditions; some *Corynebacteria* spp. are associated with cutaneous ulcer formation in susceptible individuals [28]. Taken collectively, these results imply that the changes in bacterial abundance are not the result of the oral lesions themselves, but due to the influence of smoking and SP-A levels.

**Table 3:** Bacterial species significantly altered in oral lesion samples, before and after adjustment for smoking and for SP-A levels.

				**Oral lesions only**		**Oral lesions not smoking**		**Oral lesions not SP-A**	
**Block**	**Name**	**Relative abundance (controls)**	**Relative abundance (oral lesions)**	**Log_**2**_ fold change**	**FDR *p*-value**	**Log_**2**_ fold change**	**FDR *p*-value**	**Log_**2**_ fold change**	**FDR *p*-value**
1	*Campylobacter sp._oral_taxon_044*	9.94E-03	0.00E+00	−6.64	3.0E-02	−7.28	1.0E-02		
	*Granulicatella elegans*	7.60E-03	0.00E+00	−6.79	3.0E-02	−7.44	1.0E-02		
	*Haemophilus pittmaniae*	5.00E-02	3.23E-04	−6.98	3.0E-02	−7.61	1.0E-02		
	*Haemophilus sp._oral_taxon_036*	9.10E-03	0.00E+00	−6.5	3.0E-02	−7.14	1.0E-02		
	*Neisseria shayeganii*	6.36E-03	0.00E+00	−6.67	3.0E-02	−7.31	2.0E-02		
	*Streptococcus rubneri*	4.15E-02	2.34E-03	−6.47	3.0E-02	−7.02	1.0E-02		
	*Veillonella sp._oral_taxon_780*	2.08E-02	0.00E+00	−7.44	3.0E-02	−8.04	1.0E-02		
2	*Bifidobacterium dentium*	4.09E-02	0.00E+00	−9.3	2.0E-02			8.03	9.3E-03
3	*Actinomyces israelii*	3.95E-03	0.00E+00	−5.87	4.0E-02				
	*Ruminococcaceae_[G-1] sp._oral_taxon_075*	4.31E-03	0.00E+00	−5.63	4.0E-02				
	*Prevotella shahii*	0.00E+00	3.18E-03	5.74	4.0E-02				
	*Ottowia sp._oral_taxon_894*	0.00E+00	5.55E-03	5.84	4.0E-02				
	*Stomatobaculum longum*	1.09E-04	1.15E-02	5.9	4.0E-02				
	*Leptotrichia sp._oral_taxon_392*	0.00E+00	8.92E-03	6.37	3.0E-02				
	*Capnocytophaga granulosa*	0.00E+00	1.00E-02	6.76	3.0E-02				
	*Capnocytophaga gingivalis*	0.00E+00	1.06E-02	6.81	3.0E-02				

*OTU best-matches are shown in the “Name” column. The next two columns display the relative abundance for control and oral lesions groups, with the higher value in blue and the lower value in green. The next two columns show the fold difference between lesions and controls, and the FDR p-value, with no removal of confounders. The final four columns identify if there are significant differences between oral lesions and controls after accounting for smoking and SP-A levels. The results fall in 3 blocks. Block 1 shows bacterial OTUs that are confounded by SP-A levels. Block 2 shows bacterial OTUs that are confounded by smoking. Block 3 shows OTUs confounded by smoking and SP-A levels*.

**Table 4:** Bacteria sensitive to salivary SP-A levels.

**Name**	**Log_**2**_ fold change**	**FDR *p*-value**
*Corynebacterium argentoratense*	−11.65	3.27E-05
*Streptococcus sp._oral_taxon_057*	−10.08	4.42E-04
*Stomatobaculum longum*	−8.62	9.96E-04
*Leptotrichia trevisanii*	−8.46	1.93E-03
*Prevotella albensis*	−8.28	9.96E-04
*Capnocytophaga granulosa*	−8.26	8.56E-04
*Propionibacterium propionicum*	−8.25	9.96E-04
*Capnocytophaga gingivalis*	−8.16	1.93E-03
*Catonella morbi*	−8.01	1.36E-03
*Streptococcus lactarius*	−7.93	4.42E-04
*Leptotrichia sp._oral_taxon_392*	−7.74	1.93E-03
*Lachnoanaerobaculum orale*	−7.64	3.23E-03
*Ottowia sp._oral_taxon_894*	−7.26	4.78E-03
*Prevotella shahii*	−7.07	4.67E-03
*Actinomyces odontolyticus*	−6.93	1.93E-03
*Neisseria elongata*	−6.84	1.93E-03
*Kingella denitrificans*	−6.75	5.10E-03
*Selenomonas sp._oral_taxon_137*	−6.69	8.43E-03
*Oribacterium asaccharolyticum*	−6.44	4.93E-03
*Capnocytophaga leadbetteri*	−6.43	1.00E-02
*Leptotrichia sp._oral_taxon_221*	−6.34	1.00E-02
*Treponema socranskii*	−6.32	8.98E-03
*Actinomyces sp._oral_taxon_448*	−6.19	3.47E-03
*Streptococcus anginosus*	−6.14	4.40E-03
*Cardiobacterium valvarum*	−5.83	1.00E-02
*Streptococcus vestibularis*	−5.66	8.09E-03
*Actinomyces oris*	−5.63	8.98E-03
*Selenomonas noxia*	−5.39	1.00E-02
*Actinomyces johnsonii*	−5.06	1.00E-02
*Neisseria subflava*	−4.83	1.00E-02
*Streptococcus sinensis*	6.32	4.42E-04
*Aggregatibacter sp._oral_taxon_458*	6.89	2.90E-03
*Haemophilus pittmaniae*	6.91	1.00E-02
*Haemophilus paraphrohaemolyticus*	8.04	7.23E-03
*Neisseria perflava*	10.86	3.27E-05

*Bacteria with a FDR-adjusted p-value < 0.01 are shown. The table is sorted by response to SP-A levels, with bacteria with an inverse relationship to SP-A shown with negative values*.

### Biomarkers in Microbiome Data

**Figure 3:**
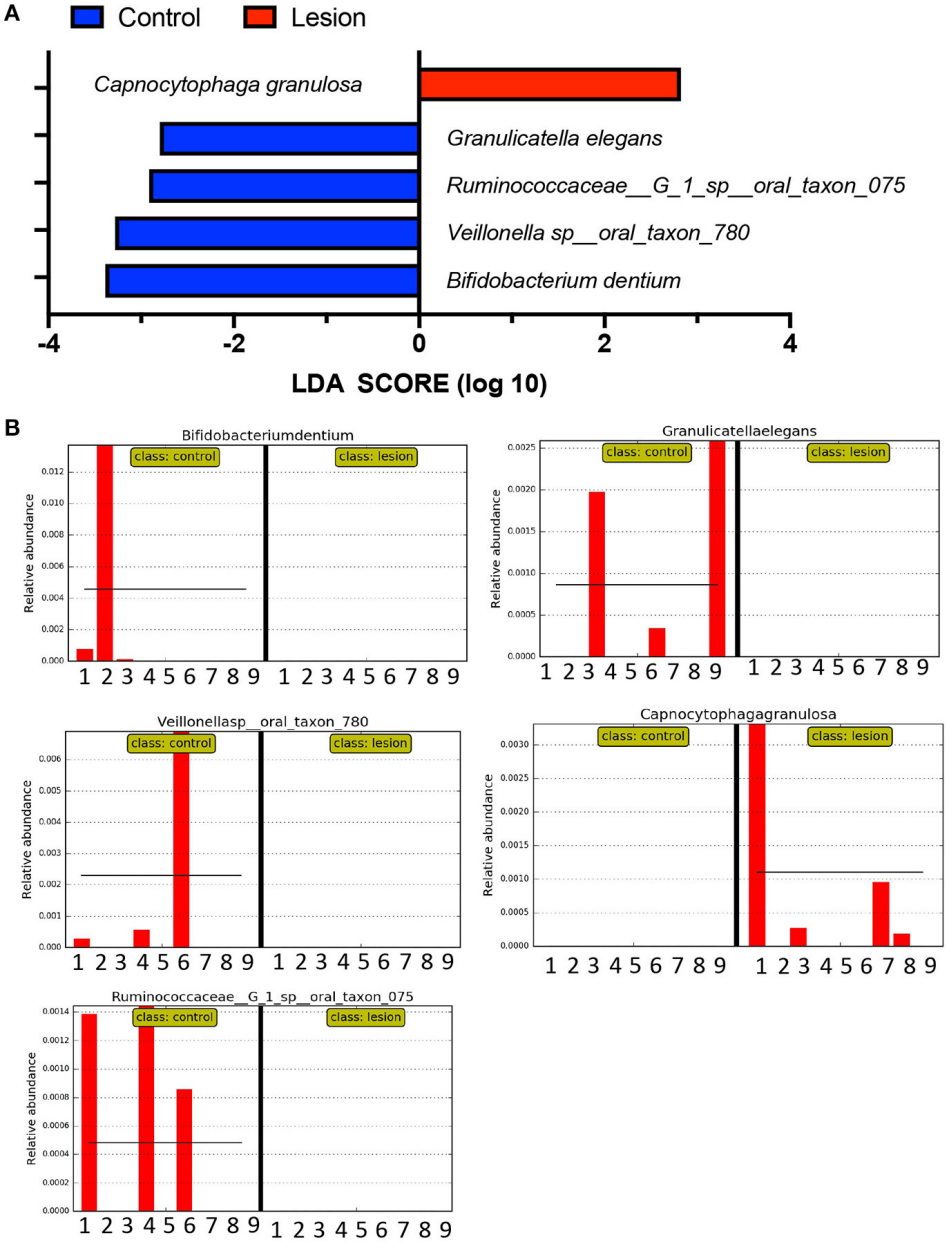
Linear discriminant analysis of microbiome data. Using LefSe, we queried our 18 microbiome samples to identify OTUs most likely to define the oral lesion condition, using an LDA cutoff of two orders of magnitude. **(A)** Collectively, one bacterial species was identified as enriched in oral lesion subjects, *C. granulosa*, shown in red. Four bacterial species were elevated in controls compared to oral lesion patients and are shown in blue. **(B)** The distribution of potential biomarkers by subject. For each chart, the first nine columns represent controls, while the second nine columns represent oral lesion subjects. At least one of the four control markers is found in six of nine subjects, and the oral lesion marker is found in four of the nine patients.

The differential abundance data indicates differences between the oral lesion group and controls, primarily driven by SP-A levels and smoking status. We submitted our microbiome community data set to LEfSe, to determine if linear discriminant analysis could identify biomarker candidates. Six species were highlighted as potential biomarkers (Figure 3). One species, *Capnocytophaga granulosa*, was elevated in four oral lesion patients but absent from all controls. The remaining four species were elevated in healthy controls and absent in oral lesion patients. One biomarker continued to be significant for discriminating oral lesion patients from smokers, with *Bifidobacterium dentium* being depleted in oral lesion patients regardless of smoking status. There were no biomarkers for oral lesion status that were independent of SP-A level. Although this is a pilot study with microbiome data from only 18 subjects, these results indicate the need for further investigation of SP-A and microbiome data as potential markers for oral lesion risk.

## Discussion

This preliminary study sought identify potential correlations between oral lesion risk factors, SP-A and microbiome composition, in an effort to better understand the early events that may underly oral lesion formation. Some aspects of this pilot study should be considered when interpreting the data, including the small number of subjects, the early and mild state of the disease, the expected high levels of inter-individual variability in the salivary microbiome, and the low number of smokers in the microbiome sequencing arm of the study. Further, there may be other host factors such as diet, oral hygiene habits, and recent use of antibiotics or antimicrobials that could influence outcomes and the precision of the use of specific bacteria as biomarkers. Regardless, this is the first study to report the influence of SP-A levels on oral microbiome composition, an observation that further underscores the interplay between host immune status and bacterial colonization. Identifying salivary SP-A as a host immune factor that may predispose to inflammation or infection may have implications beyond risk of oral ulceration.

Saliva is an ideal biological sample for risk screening, as it is readily accessible, and the composition is influenced by changes in host health. Saliva also contains bacterial species representative of oral surfaces, and these bacterial communities are responsive to changes in local and systemic host physiology [29–31]. The identification of risk factors and biomarkers in saliva could be useful for future efforts to develop better prevention approaches. The oral lesion subjects in this study had relatively minor disease, with localized redness (Grade I) or a few localized ulcers (Grade II). Consistent with previous studies, our intraoral lesion patients are more likely to be female and ages >60 [2, 20, 32]. Our oral lesion patients were also more likely to be experiencing oral pain, and to exhibit signs of bruxism. Bruxism, or tooth grinding, can be a measure of systemic stress, and this finding should be further investigated to determine if BSI can be used as another biomarker for oral ulcer risk.

Microbiome analysis was conducted in this study to further assess the role of microorganisms and their possible associations with oral lesions. We hypothesized that alterations in the mucosal cell membrane during inflammation and ulceration will induce changes in the oral microflora population. We are basing this on the fact that the destruction of oral mucosa and/or altered salivary gland secretion can have an ecological influence on the population of the oral microflora, through habitat modification [33–36]. Our study's findings reveal that widespread oral dysbiosis is not present in these Grade I and Grade II subjects, however both SP-A and smoking significantly impact community structure, as indicated by PERMANOVA analysis. Considering the variable of oral lesions alone, select oral species are significantly altered, however all of these species are more strongly influenced by smoking, SP-A levels, or both. Considering that smoking and gender are risk factors for oral lesion formation and influence SP-A levels, this provides evidence that changes in the oral microbiome detected here may directly result from the risk factors and not from the habitat change induced by the oral lesions. Further exploration and evidence would be required to definitively determine if the bacterial changes precede lesion formation.

The SP-A is a water-soluble protein and contains functional carbohydrate-recognition domains. It is part of the innate immune system and promotes the phagocytosis of bacterial cells in the lung alveoli by macrophages [37, 38]. The role of SP-A protein in saliva is unknown but it is expected to be involved in protecting the oral mucosa from foreign microbes. Our findings replicated our earlier work and showed SP-A level is significantly reduced in female smokers compared to non-smokers. Our male subjects did not have the same response, implying a gender specific mechanism of oral SP-A production and inhibition. There is a significant body of literature supporting sex-dependent regulation of and by SP-A in the pulmonary milieu. During pulmonary development, SP-A expression is regulated by a complex array of factors including hormones [39]. Further, alveolar macrophage response to infection is regulated in a sex-specific manner by Sp-A during ozone exposure [40–42]. Our findings in the oral cavity microbiome demonstrate that oral SP-A contributes to the relative proportions of bacteria in the community, whether this is through opsonization and macrophage activation is unknown but worthy of further study. There is a noticeable trend in decreasing SP-A levels in the saliva of female oral lesion subjects, and although this did not rise to the level of significance, it provides us with data for designing future studies with adequate power.

Dysbiosis induced by SP-A levels is likely an explanation for the >1,000-fold increase in *Corynebacterium argentoratense. Corynebacterium argentoratense* is commonly found in saliva and was first identified associated with tonsillitis [43]. It is also a pathogen in pharyngitis, upper respiratory infection and has been isolated from blood cultures of cancer patients [43, 44]. *Corynebacteria* in general are important opportunistic pathogens in the head and neck and upper respiratory tract, and some species are associated with cutaneous ulcers in humans and animals [28]. Future studies should further investigate SP-A interaction with oral *Corynebacteria* spp. to determine if this organism is sensitive to salivary SP-A, and to identify potential toxin production associated with oral lesion formation in otherwise healthy individuals.

The identification of one bacterial species as a potential biomarker for oral lesions, *Capnocytophaga granulosa*, is intriguing. *Capnocytophaga* spp. have been associated with inflammatory oral diseases such as periodontitis, and these bacteria are attracted to dead and dying cells to feed off of necrotic debris. Theoretically, once dysbiosis is initiated by changes in SP-A, oral pathogens such as *Corynebacteria* could stimulate the inflammatory cascades that contribute to tissue destruction of the mucosal membrane, and organisms such as *Capnocytophaga* could serve as a marker for the cellular damage [45–48]. If *Capnocytophaga* is elevated in the oral lesions, we anticipate that future studies could utilize direct lesion sampling to clarify this point.

In conclusion, this pilot study demonstrates that salivary SP-A production is gender dependent, with female reduction in average SP-A levels occurring with smoking and in the presence of oral lesions. Salivary SP-A is anticipated to target bacteria for clearance from the oral cavity, and consistent with that putative role, has a significant impact on microbiome community structure. Select microbes were identified with the potential to act as biomarkers for SP-A induced dysbiosis, however future studies will need to be performed to confirm these findings and identify additional markers to strengthen the potential of this approach. These results do contribute to the larger literature on gender-specific activities of SP-A, however the specific mechanism of action of oral SP-A, and it's specificity for oral microbes, in currently unknown. This study is important for the community of intraoral lesion patients because it provides a path of investigation for risk factors (SP-A levels) and biomarkers (bacterial changes and BSI scores) that could indicate an immunocompromised state (potential increased risk of morbidity) and association with risk for oral lesions. Understanding this phenomenon will be crucial toward developing chairside risk-assessment of patients based on simple screening data and saliva collection [49, 50].

## Ethics Statement

The studies involving human participants were reviewed and approved by IRB ethical board of the University of Texas Health Science Center at Houston (Approval number: HSC-DB-15-0742). The patients/participants provided their written informed consent to participate in this study.

## Author Contributions

SA and WF contributed to conception, design, data acquisition and analysis, drafted, and critically revised the manuscript. DS contributed to data acquisition and analysis and drafted the manuscript. GT and JA contributed to data acquisition, data analysis, figure creation, drafted the manuscript, and critically revised the manuscript. All authors contributed to the article and approved the submitted version.

## Conflict of Interest

The authors declare that the research was conducted in the absence of any commercial or financial relationships that could be construed as a potential conflict of interest.

## Supplementary Material

Supplemental Figure 1 Rarefication curves for samples included in the study, using estimated OTU. The average estimated OTU values for oral lesion subjects is not significantly different from controls.

Supplemental Table 1 Raw read data from OTUs identified and used in this study.
