# Supplementary figures and images for: Surfactant Protein A and Microbiome Composition in Patients With Atraumatic Intraoral Lesions
Source: Front Oral Health. 2021 Apr 22;2:663483. doi: 10.3389/froh.2021.663483 (PMC8757703; doi:10.3389/froh.2021.663483)

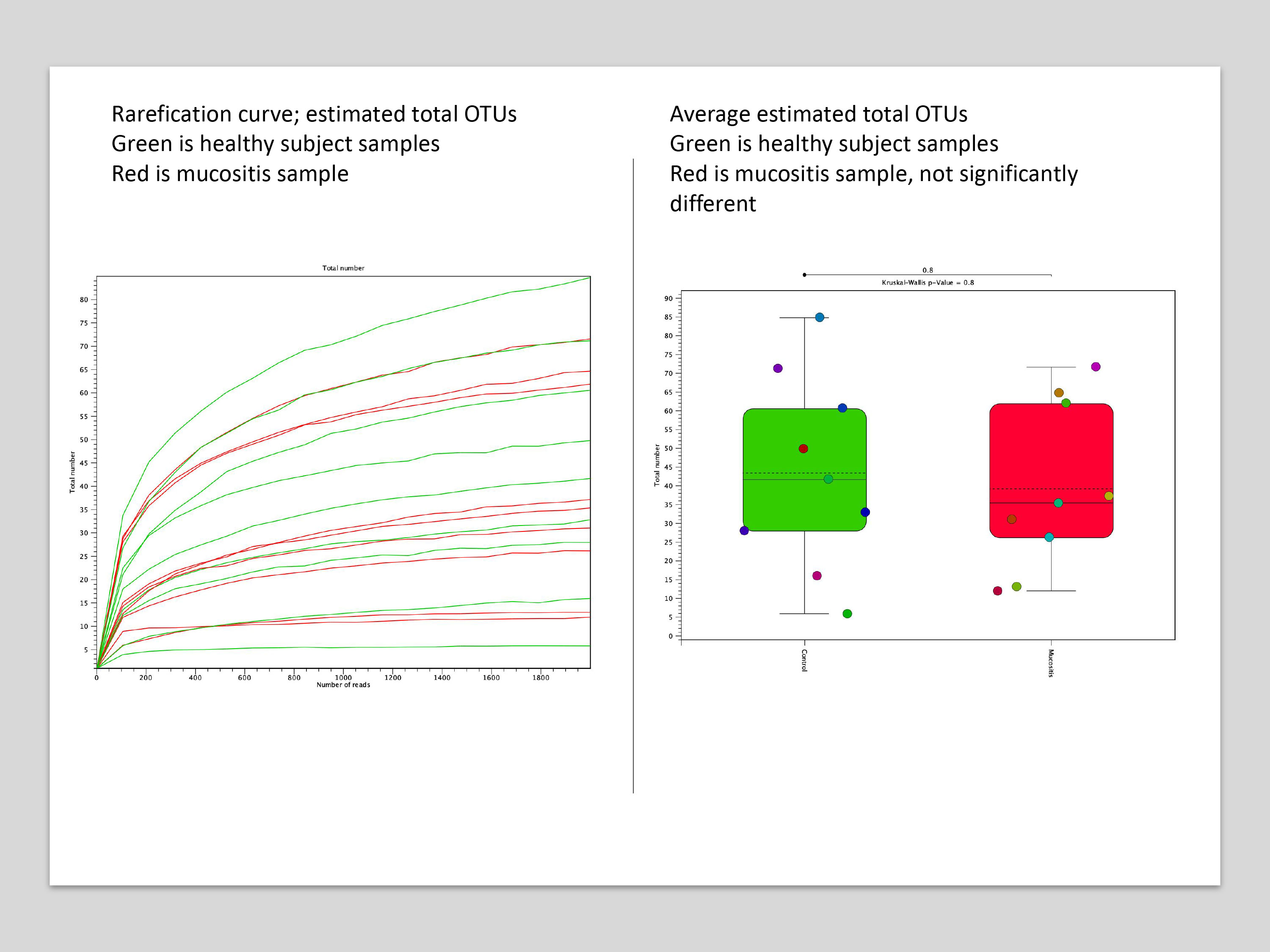

Supplement: Supplemental Figure 1 — Rarefication curves for samples included in the study, using estimated OTU. The average estimated OTU values for oral lesion subjects is not significantly different from controls. [file Image_1.JPEG]
